# Supplementary material for: Arachidic Acid-Carrying Phosphatidylglycerol Lipids Statistically Mediate the Relationship Between Central Adiposity and Cognitive Function in Cognitively Unimpaired Older Adults
Source: Nutrients. 2025 Oct 29;17(21):3405. doi: 10.3390/nu17213405 (PMC12610110; doi:10.3390/nu17213405)
Supplement: Supplementary file 1 [file nutrients-17-03405-s001.zip › nutrients-3920970-supplementary.pdf]

Arachidic Acid-Carrying Phosphatidylglycerol Lipids Statistically Mediate the Relationship Between Central Adiposity and Cognitive Function in Cognitively Unimpaired Older Adults

Maria Kadyrov [1,2,3,*], Luke Whiley [1,4,5,6], Kirk I. Erickson [7,8], Belinda Brown [2] and Elaine Holmes [1,9,*]

[1] Centre for Computational and Systems Medicine, Health Futures Institute, Murdoch University, Harry Perkins Building, Perth, WA 6150, Australia; luke.whiley@murdoch.edu.au
[2] Centre for Healthy Ageing, Health Futures Institute, Murdoch University, Perth, WA 6150, Australia; belinda.brown@murdoch.edu.au
[3] School of Health Sciences, University of Notre Dame, Perth, WA 6160, Australia
[4] Curtin Medical School, Faculty of Health Sciences, Curtin University, Perth, WA 6102, Australia
[5] Curtin Medical Research Institute (Curtin-MRI), Curtin University, Perth, WA 6102, Australia
[6] Dementia Centre of Excellence, enAble Institute, Curtin University, Perth, WA 6102, Australia
[7] AdventHealth Research Institute, Neuroscience Institute, Orlando, FL 32804, USA; kirk.erickson@adventhealth.com
[8] Department of Psychology, University of Pittsburgh, Pittsburgh, PA 15260, USA
[9] Section for Nutrition Research, Department of Metabolism, Digestion and Reproduction, Imperial College London, London SW7 2AZ, UK
* Correspondence: maria.kadyrov@murdoch.edu.au (M.K.); elaine.holmes@murdoch.edu.au (E.H.)

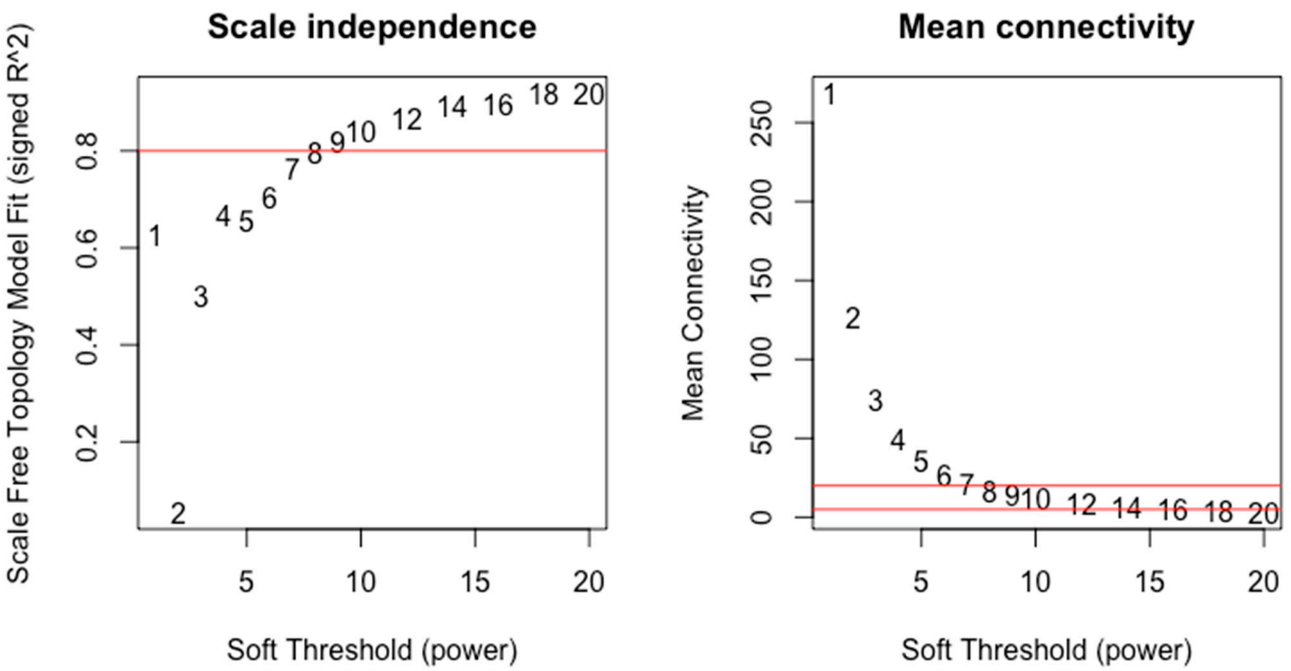

Figure S1 Selection of soft-thresholding power for WGCNA network construction. Scale-free topology fit index (left) and mean connectivity (right) are plotted across a range of soft-thresholding powers (1–20). A power of 9 was selected, corresponding to the lowest value that achieved a scale-free topology model fit of $R^2 > 0.80$ while maintaining adequate mean connectivity.

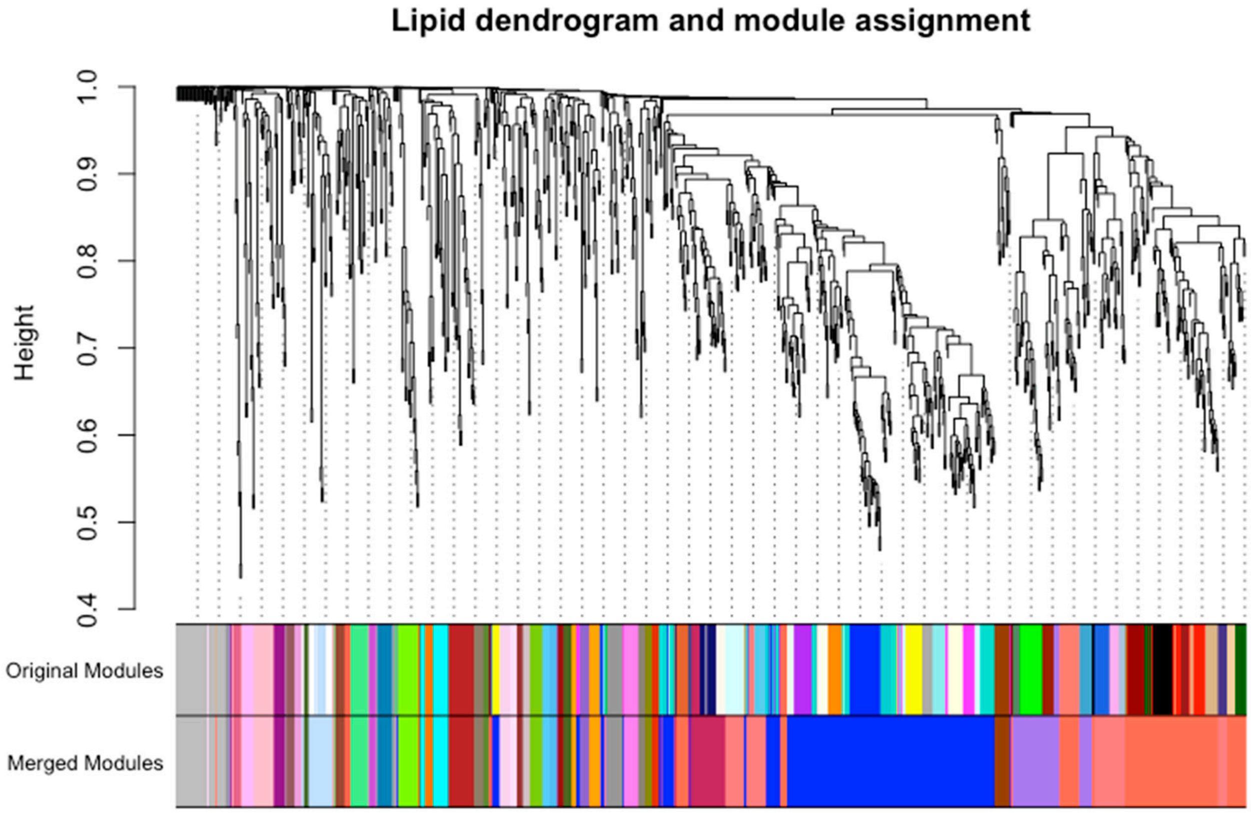

Figure S2. Lipid co-expression dendrogram and module assignment from WGCNA. Hierarchical clustering of lipid species based on topological overlap, with modules identified by dynamic tree cutting (top row). Closely related modules with eigengene correlation > 0.75 were subsequently merged (bottom row), resulting in the final set of lipid co-expression modules used for downstream analyses

Table S1. Cognitive assessment tasks contributing to each composite score
Each cognitive domain was derived from standardised neuropsychological assessments, with individual test scores aggregated to form domain-specific composite measures.

Composite	Test
Attention	WAIS-III – Digit Span (Forward only)
	Cogstate – Identification Task
Delayed Recall	CVLT-II – Long Delay Free Recall
	BVMT – Long Delay Recall
	Cogstate – Groton Maze Recall
Episodic Memory	CVLT-II – Learning, Short & Long Delay Free Recall, Recognition D`
	BVMT – Long Delay Recall
	Cogstate – Groton Maze Recall
Executive Function	NIH-EXAMINER – Phonemic Fluency, Flanker, Set-shifting
	TMT – Part B
Global Cognition	WAIS-III – Digit Span
	Cogstate – One-Back, Identification Task, Groton Maze Recall
	CVLT-II – Learning, Short & Long Delay Recall, Recognition D`
	BVMT – Learning and Long Delay Recall
	NIH-EXAMINER – Phonemic Fluency, Flanker, Set-shifting
	TMT – Part B
Learning	CVLT-II – Learning
	BVMT – Learning
Working Memory	WAIS-III – Digit Span (Back only)
	Cogstate – One-Back

Abbreviations: BVMT, Brief Visuospatial Memory Test; CVLT-II, California Verbal Learning Test; NIH-EXAMINER, The National Institutes of Health - Executive Abilities: Measures and Instruments for Neurobehavioral Evaluation and Research; TMT, Trail Making Test; WAIS-III, Wechsler Adult Intelligence Scale-III

Table S2. Lipid species composition of WGCNA modules

Each row lists the individual lipid species assigned to a given WGCNA module (m1–m39). Lipids within the same module exhibit highly correlated patterns of change across participants and are grouped according to shared structural or functional characteristics. Lipid species are separated by semicolons (;) within each cell.

Module	Lipids
m1	PC(18:0_18:3); PC(18:1_18:3); PC(18:2_18:3)PE(18:2_18:3); LPC(18:3); LPE(18:2); LPE(18:3)
m2	Cer(d18:1/18:0); Cer(d18:1/20:0); DG(14:0_14:0); DG(14:0_16:1); DG(14:0_18:1); DG(14:0_18:2); DG(14:0_18:3); DG(14:0_20:4); DG(16:0_16:1); DG(16:0_18:1); DG(16:0_18:3); DG(16:1_16:1); DG(16:1_18:0); DG(16:1_18:1); DG(18:0_18:1); DG(18:0_18:2); DG(18:0_18:3); PC(14:0_20:3); PC(14:0_20:4); PC(14:0_22:4); PC(16:0_18:3); PE(14:0_18:1); PE(16:0_16:1); PE(18:0_16:0); PE(18:0_16:1); PE(18:1_16:1); PE(O-18:0_20:3); PE(P-14/0_18:1); PG(18:0_18:2); PI(16:0_18:3); PI(18:0_18:3); LPE(14:0); LPE(16:1); LPG(16:0); LPG(18:0); LPI(20:3); LPI(20:4); MG(16:1); TG(40:0_FA14:0); TG(40:0_FA16:0); TG(42:0_FA14:0); TG(42:0_FA16:0); TG(42:1_FA14:0); TG(42:1_FA16:0); TG(42:1_FA16:1); TG(42:1_FA18:1); TG(42:2_FA18:2); TG(44:0_FA14:0); TG(44:0_FA16:0); TG(44:0_FA18:0); TG(44:1_FA14:0); TG(44:1_FA16:0); TG(44:1_FA16:1); TG(44:1_FA18:1); TG(44:2_FA14:0); TG(44:2_FA16:0); TG(44:2_FA16:1); TG(44:2_FA18:1); TG(44:2_FA18:2); TG(44:3_FA18:2); TG(45:0_FA14:0); TG(45:0_FA16:0); TG(45:1_FA16:0); TG(45:1_FA18:1); TG(46:0_FA14:0); TG(46:0_FA16:0); TG(46:0_FA18:0); TG(46:1_FA14:0); TG(46:1_FA16:0); TG(46:1_FA16:1); TG(46:1_FA18:0); TG(46:1_FA18:1); TG(46:2_FA14:0); TG(46:2_FA16:0); TG(46:2_FA16:1); TG(46:2_FA18:1); TG(46:2_FA18:2); TG(46:3_FA14:0); TG(46:3_FA16:0); TG(46:3_FA16:1); TG(46:3_FA18:1); TG(46:3_FA18:2); TG(46:3_FA18:3); TG(46:4_FA18:2); TG(47:0_FA14:0); TG(47:0_FA16:0); TG(47:0_FA17:0); TG(47:1_FA14:0); TG(47:1_FA16:0); TG(47:1_FA16:1); TG(47:1_FA17:0); TG(47:1_FA18:1); TG(47:2_FA14:0); TG(47:2_FA16:1); TG(47:2_FA18:1); TG(47:2_FA18:2); TG(48:0_FA14:0); TG(48:0_FA16:0); TG(48:0_FA18:0); TG(48:1_FA14:0); TG(48:1_FA16:0); TG(48:1_FA16:1); TG(48:1_FA18:0); TG(48:1_FA18:1); TG(48:2_FA14:0); TG(48:2_FA16:0); TG(48:2_FA16:1); TG(48:2_FA18:0); TG(48:2_FA18:1); TG(48:2_FA18:2); TG(48:3_FA14:0); TG(48:3_FA16:0); TG(48:3_FA16:1); TG(48:3_FA18:1); TG(48:3_FA18:2); TG(48:3_FA18:3); TG(48:4_FA14:0); TG(48:4_FA16:0); TG(48:4_FA16:1); TG(48:4_FA18:1); TG(48:4_FA18:3); TG(48:4_FA20:4); TG(49:0_FA16:0); TG(49:0_FA17:0); TG(49:0_FA18:0); TG(49:1_FA14:0); TG(49:1_FA16:0); TG(49:1_FA16:1); TG(49:1_FA17:0); TG(49:1_FA18:1); TG(49:2_FA14:0); TG(49:2_FA16:0); TG(49:2_FA16:1); TG(49:2_FA17:0); TG(49:2_FA18:1); TG(49:2_FA18:2); TG(49:3_FA16:0); TG(49:3_FA16:1); TG(49:3_FA18:3); TG(50:0_FA14:0); TG(50:0_FA16:0); TG(50:0_FA18:0); TG(50:1_FA14:0); TG(50:1_FA16:0); TG(50:1_FA16:1); TG(50:1_FA18:0); TG(50:1_FA18:1); TG(50:1_FA20:1); TG(50:2_FA16:0); TG(50:2_FA16:1); TG(50:2_FA18:0); TG(50:2_FA18:1); TG(50:2_FA18:2); TG(50:2_FA20:2); TG(50:3_FA16:0); TG(50:3_FA16:1); TG(50:3_FA18:0); TG(50:3_FA18:3); TG(50:3_FA20:3); TG(50:4_FA16:0); TG(50:4_FA18:1); TG(50:4_FA20:4); TG(50:5_FA16:0); TG(50:5_FA18:1); TG(50:5_FA20:4); TG(50:6_FA20:4); TG(51:0_FA16:0); TG(51:0_FA17:0); TG(51:0_FA18:0); TG(51:1_FA16:0); TG(51:1_FA17:0); TG(51:1_FA18:0); TG(51:1_FA18:1); TG(51:2_FA16:0); TG(51:2_FA16:1); TG(51:2_FA17:0); TG(51:2_FA18:1); TG(51:2_FA18:2); TG(51:3_FA16:1); TG(51:3_FA17:0); TG(51:3_FA18:3); TG(51:4_FA20:4); TG(52:0_FA16:0); TG(52:0_FA18:0); TG(52:0_FA20:0); TG(52:1_FA16:0); TG(52:1_FA16:1); TG(52:1_FA18:0); TG(52:1_FA18:1); TG(52:1_FA20:0); TG(52:1_FA20:1); TG(52:2_FA14:0); TG(52:2_FA16:1); TG(52:2_FA18:0); TG(52:2_FA18:2); TG(52:2_FA20:0); TG(52:2_FA20:1); TG(52:3_FA14:0); TG(52:3_FA18:0); TG(52:3_FA18:3); TG(52:3_FA20:1); TG(52:4_FA18:0); TG(52:4_FA20:2); TG(52:4_FA22:4); TG(52:5_FA14:0); TG(52:7_FA16:0); TG(52:8_FA16:1); TG(53:0_FA16:0); TG(53:1_FA16:0); TG(53:1_FA17:0); TG(53:1_FA18:0); TG(53:1_FA18:1); TG(53:2_FA17:0); TG(53:2_FA18:1); TG(53:2_FA18:2); TG(53:4_FA16:0); TG(53:4_FA20:4); TG(54:0_FA16:0); TG(54:0_FA18:0); TG(54:1_FA16:0); TG(54:1_FA18:0); TG(54:1_FA20:0); TG(54:2_FA18:2); TG(54:2_FA20:0); TG(54:2_FA20:2); TG(54:3_FA18:2); TG(54:5_FA22:5); TG(55:1_FA16:0); TG(55:1_FA18:1); TG(55:2_FA18:1); TG(55:2_FA18:2); TG(56:1_FA16:0); TG(56:1_FA18:1); TG(56:2_FA20:0); TG(56:4_FA20:4)
m3	DG(16:0_20:3); DG(16:0_20:4); DG(16:1_20:4); DG(18:1_20:2); DG(18:1_20:3); DG(18:1_20:4); DG(18:1_22:4); DG(18:2_20:4); MG(20:4); TG(50:4_FA20:3); TG(52:3_FA20:2); TG(52:3_FA20:3); TG(52:4_FA14:0); TG(52:4_FA20:3); TG(52:4_FA20:4); TG(52:5_FA20:3); TG(52:5_FA20:4); TG(52:6_FA20:4); TG(53:5_FA20:4); TG(53:6_FA20:4); TG(54:3_FA16:0); TG(54:3_FA18:0); TG(54:3_FA20:1); TG(54:3_FA20:2); TG(54:3_FA20:3); TG(54:4_FA16:0); TG(54:4_FA16:1); TG(54:4_FA20:2); TG(54:4_FA20:3); TG(54:4_FA20:4); TG(54:4_FA22:4); TG(54:5_FA16:0); TG(54:5_FA16:1); TG(54:5_FA20:2); TG(54:5_FA20:3); TG(54:5_FA20:4); TG(54:5_FA22:4); TG(54:6_FA16:1); TG(54:6_FA20:3); TG(54:6_FA20:4); TG(54:7_FA20:4); TG(54:8_FA20:4); TG(55:5_FA20:4); TG(56:3_FA16:0); TG(56:3_FA20:2); TG(56:4_FA18:0); TG(56:4_FA18:1); TG(56:4_FA20:2); TG(56:4_FA20:3); TG(56:5_FA16:0); TG(56:5_FA18:0); TG(56:5_FA18:2); TG(56:5_FA20:2); TG(56:5_FA20:3); TG(56:5_FA20:4); TG(56:5_FA22:4); TG(56:6_FA18:2); TG(56:6_FA18:3); TG(56:6_FA20:2); TG(56:6_FA20:3); TG(56:6_FA20:4); TG(56:6_FA22:4); TG(56:7_FA18:3); TG(56:7_FA20:3); TG(56:7_FA20:4); TG(56:7_FA22:4); TG(56:8_FA18:3); TG(56:8_FA20:4); TG(56:9_FA20:4); TG(58:10_FA20:4); TG(58:5_FA18:1); TG(58:6_FA20:4); TG(58:6_FA22:4); TG(58:7_FA20:4); TG(58:7_FA22:4); TG(58:8_FA20:3); TG(58:8_FA20:4); TG(58:9_FA20:4)
m4	PE(O-16:0_22:4); PE(O-18:0_22:4); PE(P-16:0_22:4); PE(P-18:0_22:4); PE(P-18:1_22:4); PG(20:0_22:4)

m5	PE(14:0_18:2); PE(16:0_18:2); PE(16:0_18:3); PE(18:0_18:0); PE(18:0_18:1); PE(18:0_18:2); PE(18:0_18:3); PE(18:1_18:1); PE(18:1_18:2); PE(18:1_18:3); PE(18:2_18:2); PE(18:2_20:1); PE(18:2_20:2); PE(O-18:0_18:1); PE(O-18:0_18:2); PE(O-18:0_18:3); PG(16:0_18:2)
m6	PC(16:0_18:1); PC(16:0_20:1); PC(18:0_18:0); PC(18:0_18:1); PC(18:0_20:1); PC(18:1_18:1); PC(18:1_20:1); PC(20:0_18:1); PC(20:0_20:2); PE(P-16:0_16:0); PE(P-16:0_18:0); PG(18:1_18:1); CE(18:3); LPI(18:0)
m7	LPC(16:0); LPC(18:0); LPC(18:1); LPC(18:2); LPC(20:0); LPC(20:1); LPC(20:2); LPC(22:5); LPE(16:0); LPE(18:0); LPE(18:1); LPE(20:0)
m8	DG(16:0_18:2); DG(16:0_22:5); DG(16:1_18:2); DG(16:1_18:3); DG(16:1_20:2); DG(18:1_18:2); DG(18:1_22:5); DG(18:2_18:3); DG(18:2_20:3); DG(18:2_22:4); PG(16:0_20:2); PG(18:0_20:2); CE(24:1); MG(18:2); MG(18:3); MG(20:3); TG(48:4_FA18:2); TG(48:5_FA18:2); TG(48:5_FA18:3); TG(49:3_FA18:2); TG(50:3_FA14:0); TG(50:3_FA18:1); TG(50:3_FA18:2); TG(50:4_FA14:0); TG(50:4_FA16:1); TG(50:4_FA18:2); TG(50:4_FA18:3); TG(50:5_FA14:0); TG(50:5_FA16:1); TG(50:5_FA18:2); TG(50:5_FA18:3); TG(51:3_FA18:2); TG(51:4_FA16:1); TG(51:4_FA18:2); TG(51:4_FA18:3); TG(51:5_FA18:2); TG(51:5_FA18:3); TG(52:3_FA16:0); TG(52:3_FA18:2); TG(52:3_FA20:0); TG(52:4_FA16:0); TG(52:4_FA16:1); TG(52:4_FA18:1); TG(52:4_FA18:2); TG(52:4_FA18:3); TG(52:4_FA20:0); TG(52:5_FA16:0); TG(52:5_FA16:1); TG(52:5_FA18:1); TG(52:5_FA18:2); TG(52:5_FA18:3); TG(52:6_FA16:1); TG(52:6_FA18:2); TG(52:6_FA18:3); TG(52:8_FA18:2); TG(53:3_FA17:0); TG(53:3_FA18:2); TG(53:4_FA17:0); TG(53:4_FA18:2); TG(53:4_FA18:3); TG(54:4_FA18:0); TG(54:4_FA18:1); TG(54:4_FA18:2); TG(54:4_FA18:3); TG(54:4_FA20:1); TG(54:5_FA18:0); TG(54:5_FA18:1); TG(54:5_FA18:2); TG(54:5_FA18:3); TG(54:6_FA16:0); TG(54:6_FA18:1); TG(54:6_FA18:2); TG(54:6_FA18:3); TG(54:7_FA18:1); TG(54:7_FA18:2); TG(54:7_FA18:3); TG(54:8_FA18:2); TG(54:8_FA18:3); TG(55:4_FA18:2); TG(55:5_FA18:1); TG(55:5_FA18:2); TG(56:3_FA18:2); TG(56:3_FA20:0); TG(56:4_FA18:2); TG(56:5_FA18:1); TG(56:5_FA20:1); TG(56:5_FA22:5); TG(56:6_FA16:0); TG(56:6_FA18:1); TG(56:7_FA16:1); TG(56:7_FA18:1); TG(57:3_FA18:2); TG(58:6_FA16:0); TG(58:6_FA18:1); TG(58:7_FA16:0); TG(58:7_FA18:1)
m9	PC(14:0_14:0); PC(14:0_18:1); PC(14:0_18:2); PC(14:0_18:3); PC(14:0_20:2); PC(14:0_22:5); PC(14:0_22:6); PC(16:0_14:0); PC(18:0_14:0); PG(18:1_18:3); PI(14:0_18:1); PS(20:0_18:3); LPC(14:0)
m10	PC(16:0_20:4); PC(18:0_20:4); PC(18:1_20:4); PC(18:2_20:4); PC(20:0_20:4); PE(O-18:0_20:4); PG(18:0_20:0); PG(18:1_20:4); PS(20:0_20:4)
m11	PC(16:0_16:0); PC(16:0_18:0); PC(16:0_22:5); PC(18:0_22:5); PC(18:1_22:5); PC(18:2_22:5); PC(20:0_22:5); PS(20:0_22:5)
m12	Cer(d18:1/14:0); PC(14:1_14:1); PC(16:0_18:2); PC(16:1_18:2); PC(18:0_18:2); PC(18:1_18:2); PC(18:2_16:1); PC(18:2_18:2); PC(18:2_20:1); PC(18:2_20:2); PE(14:0_22:6); PE(P-16:1_18:1); PG(18:1_18:2); PG(18:2_16:1); PG(18:2_18:2); PI(16:0_18:2); PI(18:0_18:2); PI(18:1_18:2); PI(18:2_16:1); PI(18:2_18:2); PS(14:0_18:2); PS(16:0_16:1); PS(16:0_18:2); PS(20:0_18:2); CE(18:2); LPI(18:2)
m13	PC(14:0_20:5); PC(16:0_20:5); PC(18:0_20:5); PC(18:1_20:5); PC(18:2_20:5); PE(16:0_20:5); PE(18:0_20:5); PE(18:1_20:5); PE(P-16:0_20:5); PE(P-18:0_20:5); PE(P-18:1_20:5); PG(18:1_20:5); PG(20:0_20:5); PG(20:0_22:5); PI(18:0_20:5); PS(18:0_20:0); PS(20:0_20:5); CE(20:5); FA(20:5); FA(22:6); LPC(20:5); LPE(20:5)
m14	DG(14:0_20:0); DG(16:0_16:0); DG(16:1_20:0); DG(20:0_20:0); DhCer(d18:0/16:0); DhCer(d18:0/26:0); HexCer(d18:1/20:1); PC(18:2_22:4); PE(O-16:0_16:0); PE(O-16:0_20:1); PE(O-16:0_20:2); PE(O-18:0_18:0); PE(O-18:0_20:1); PG(14:0_18:1); PG(18:0_20:4); PG(18:1_22:5); PG(18:2_20:4); PI(18:2_20:1); PS(16:0_20:4); PS(18:0_20:4); PS(18:1_18:2); PS(20:0_20:1); CE(14:0); CE(16:0); CE(20:0); CE(20:1); CE(20:2); CE(20:4); CE(22:1); CE(22:2); CE(22:4); FA(24:0); LPE(20:1); LPE(20:2); LPG(18:2); MG(14:0); MG(16:0); MG(18:0); MG(22:5); TG(55:3_FA18:2); TG(56:4_FA16:0); TG(56:4_FA22:4); TG(57:10_FA22:6)
m15	PC(16:0_22:6); PC(18:0_22:6); PC(18:1_22:6); PC(18:2_22:6); PC(20:0_22:6); PI(16:0_22:6); PI(18:0_18:0); PI(18:0_20:0); PI(18:0_22:6); PI(20:0_16:1); PI(20:0_18:1); PI(20:0_18:2); PI(20:0_20:4); PS(20:0_22:6); LPC(22:6); LPE(22:6)
m16	Cer(d18:1/20:1); Cer(d18:1/22:1); HexCer(d18:1/18:1); HexCer(d18:1/22:1); PG(18:2_20:1)
m17	PE(O-16:0_22:6); PE(O-18:0_22:6); PE(P-16:0_22:6); PE(P-18:0_22:6); PE(P-18:1_22:6); PE(P-18:2_22:6); PG(20:0_22:6)
m18	PI(16:0_20:3); PI(16:0_20:4); PI(16:0_22:4); PI(18:0_20:4); PI(18:0_22:4)
m19	DG(14:0_22:6); DG(16:0_20:5); DG(16:0_22:6); DG(16:1_22:6); DG(18:1_20:5); DG(18:1_22:6); DG(18:2_20:5); DG(18:2_22:5); DG(18:2_22:6); CE(22:5); CE(22:6); TG(50:5_FA20:5); TG(52:5_FA20:5); TG(52:5_FA22:5); TG(52:6_FA14:0); TG(52:6_FA16:0); TG(52:6_FA18:1); TG(52:6_FA20:5); TG(52:6_FA22:6); TG(52:7_FA18:1); TG(52:7_FA20:5); TG(52:7_FA22:6); TG(54:5_FA20:5); TG(54:6_FA20:5); TG(54:6_FA22:5); TG(54:6_FA22:6); TG(54:7_FA16:1); TG(54:7_FA20:5); TG(54:7_FA22:5); TG(54:7_FA22:6); TG(54:8_FA20:5); TG(54:8_FA22:6); TG(55:7_FA22:6); TG(56:6_FA18:0); TG(56:6_FA20:5); TG(56:6_FA22:5); TG(56:6_FA22:6); TG(56:7_FA16:0); TG(56:7_FA18:0); TG(56:7_FA18:2); TG(56:7_FA20:5); TG(56:7_FA22:5); TG(56:7_FA22:6); TG(56:8_FA16:0); TG(56:8_FA16:1); TG(56:8_FA18:1); TG(56:8_FA18:2); TG(56:8_FA20:5); TG(56:8_FA22:5); TG(56:8_FA22:6); TG(56:9_FA18:3); TG(56:9_FA20:5); TG(56:9_FA22:6); TG(58:10_FA18:2); TG(58:10_FA20:5); TG(58:10_FA22:5); TG(58:10_FA22:6); TG(58:6_FA18:0); TG(58:6_FA22:5);

	TG(58:7_FA18:2); TG(58:7_FA22:5); TG(58:7_FA22:6); TG(58:8_FA18:1); TG(58:8_FA18:2); TG(58:8_FA22:5); TG(58:8_FA22:6); TG(58:9_FA18:1); TG(58:9_FA18:2); TG(58:9_FA22:5); TG(58:9_FA22:6); TG(60:10_FA22:5); TG(60:10_FA22:6); TG(60:11_FA22:5); TG(60:11_FA22:6)
m20	PI(16:0_14:0); PI(16:0_16:0); PI(16:0_16:1); PI(16:0_18:0); PI(16:0_18:1); PI(16:0_20:2); PI(16:0_22:5); PI(18:0_16:1); PI(18:0_18:1); PI(18:0_22:5); PI(18:1_16:1); PI(18:1_18:1); PI(18:1_22:5); LPI(16:0); LPI(16:1); LPI(18:1)
m21	PG(20:0_16:1); PG(20:0_18:1); PG(20:0_18:2); PG(20:0_20:1); PG(20:0_20:2)
m22	PC(16:0_20:2); PC(18:0_20:2); PC(18:1_20:2); PG(18:1_20:2); PI(18:0_20:2); PI(18:0_20:3); PI(18:1_20:2); PI(18:1_20:3); PI(18:1_20:4); PI(18:2_20:4)
m23	DG(18:1_18:1); DG(18:1_20:1); MG(18:1); MG(22:4); TG(50:2_FA14:0); TG(52:2_FA16:0); TG(52:2_FA18:1); TG(52:3_FA16:1); TG(52:3_FA18:1); TG(53:3_FA16:0); TG(54:1_FA18:1); TG(54:1_FA20:1); TG(54:2_FA16:0); TG(54:2_FA18:0); TG(54:2_FA18:1); TG(54:2_FA20:1); TG(54:3_FA16:1); TG(54:3_FA18:1); TG(55:4_FA18:1); TG(56:2_FA16:0); TG(56:2_FA18:0); TG(56:2_FA20:1); TG(56:3_FA18:0); TG(56:3_FA18:1); TG(56:3_FA20:1); TG(57:2_FA18:1); TG(58:2_FA18:1); TG(58:3_FA18:1)
m24	PE(16:0_22:6); PE(18:0_22:6); PE(18:1_22:6); PS(18:0_18:0); PS(18:0_18:1); PS(18:0_18:2)
m25	LPC(20:3); LPC(20:4); LPC(22:4); LPE(20:3); LPE(20:4)
m26	PC(16:0_20:3); PC(18:0_20:3); PC(18:1_20:3); PC(18:2_20:3); PC(20:0_20:3); PG(18:1_20:3); PG(18:2_20:3); PS(20:0_20:2); PS(20:0_20:3); CE(20:3)
m27	PE(16:0_16:0); PE(16:0_18:1); PE(16:0_20:3); PE(16:0_20:4); PE(16:0_22:4); PE(16:0_22:5); PE(18:0_20:3); PE(18:0_20:4); PE(18:0_22:4); PE(18:0_22:5); PE(18:1_20:3); PE(18:1_20:4); PE(18:1_22:4); PE(18:1_22:5); PE(18:2_20:3); PE(18:2_20:4); PG(16:0_18:1)
m28	PE(O-16:0_20:4); PE(O-16:0_20:5); PE(P-16:0_20:4); PE(P-18:0_20:4); PE(P-18:1_20:4); PE(P-18:2_20:4); PG(20:0_20:4)
m29	PC(16:0_16:1); PC(16:1_18:1); PC(18:0_16:1); PE(O-18:0_16:1); PG(18:1_16:1); PS(20:0_16:1); PS(20:0_18:1); CE(16:1); LPC(16:1); LPG(18:1)
m30	HexCer(d18:1/16:0); HexCer(d18:1/18:0); HexCer(d18:1/20:0); HexCer(d18:1/22:0); HexCer(d18:1/24:0); HexCer(d18:1/24:1); HexCer(d18:1/26:0); HexCer(d18:1/26:1)
m31	PE(16:0_20:1); PE(16:0_20:2); PE(18:0_20:1); PE(18:0_20:2); PE(18:1_20:1); PE(18:1_20:2)
m32	Cer(d18:1/22:0); Cer(d18:1/24:0); Cer(d18:1/24:1); Cer(d18:1/26:0); Cer(d18:1/26:1); DhCer(d18:0/18:0); DhCer(d18:0/20:0); DhCer(d18:0/22:0); DhCer(d18:0/22:1); DhCer(d18:0/24:0); DhCer(d18:0/24:1); CE(18:0); CE(18:1)
m33	FA(14:0); FA(14:1); FA(16:0); FA(16:1); FA(16:2); FA(18:0); FA(18:1); FA(18:2); FA(18:3); FA(20:0); FA(20:1); FA(20:2); FA(20:3); FA(20:4); FA(22:4); FA(22:5); FA(24:1)
m34	PE(18:2_16:1); PE(O-16:0_18:2); PE(O-16:0_18:3); PE(P-16:0_18:2); PE(P-16:0_18:3); PE(P-18:0_18:2); PE(P-18:0_18:3); PE(P-18:1_18:2); PE(P-18:1_18:3); PE(P-18:2_18:2); PG(20:0_18:3)
m35	Cer(d18:1/16:0); DhCer(d18:0/26:1); SM(d18:1/14:0); SM(d18:1/16:0); SM(d18:1/18:0); SM(d18:1/18:1); SM(d18:1/20:1); SM(d18:1/22:0); SM(d18:1/22:1); SM(d18:1/24:0); SM(d18:1/24:1); SM(d18:1/26:0); SM(d18:1/26:1)
m36	HexCer(d18:1/14:0); LacCer(d18:1/14:0); LacCer(d18:1/16:0); LacCer(d18:1/24:0); LacCer(d18:1/24:1); PE(O-18:0_16:0)
m37	PC(18:0_20:0); PE(O-16:0_16:1); PE(O-16:0_22:5); PE(O-18:0_22:5); PE(P-16:0_16:1); PE(P-16:0_22:5); PE(P-18:0_16:1); PE(P-18:0_22:5); PE(P-18:1_16:1); PE(P-18:1_22:5)
m38	PE(O-16:0_18:1); PE(O-16:0_20:3); PE(P-16:0_18:1); PE(P-16:0_20:1); PE(P-16:0_20:2); PE(P-16:0_20:3); PE(P-18:0_16:0); PE(P-18:0_18:0); PE(P-18:0_18:1); PE(P-18:0_20:1); PE(P-18:0_20:2); PE(P-18:0_20:3); PE(P-18:1_16:0); PE(P-18:1_18:0); PE(P-18:1_18:1); PE(P-18:1_20:1); PE(P-18:1_20:2); PE(P-18:1_20:3); PG(20:0_20:3)
m39	PC(16:0_22:4); PC(18:0_22:4); PC(18:1_22:4); PC(20:0_22:4); PG(18:1_22:4); PS(20:0_22:4); LPE(22:4); LPE(22:5)

Abbreviations: CE, Cholesteryl Ester; Cer, Ceramide; DG, Diacylglycerol; DhCer, Dehydroxyceramide; FA, Free Fatty Acid; HexCer, Hexosylceramide; LacCer, Lactosylceramide; LPC, Lysophosphatidylcholine; LPE, Lysophosphatidylethanolamine; LPG, Lysophosphatidylglycerol; LPI, Lysophosphatidylinostol; MG, Monoacylglycerol; PC, Phosphatidylcholine; PE, Phosphatidylethanolamine; PE.P, Plasmenyl-PE; PE.O, Plasmanyl-PE; PG, Phosphatidylglycerol; PI, Phosphatidylinositol; PS, Phosphatidylserine; SM, Sphingomyelin; TG, Triacylglycerol

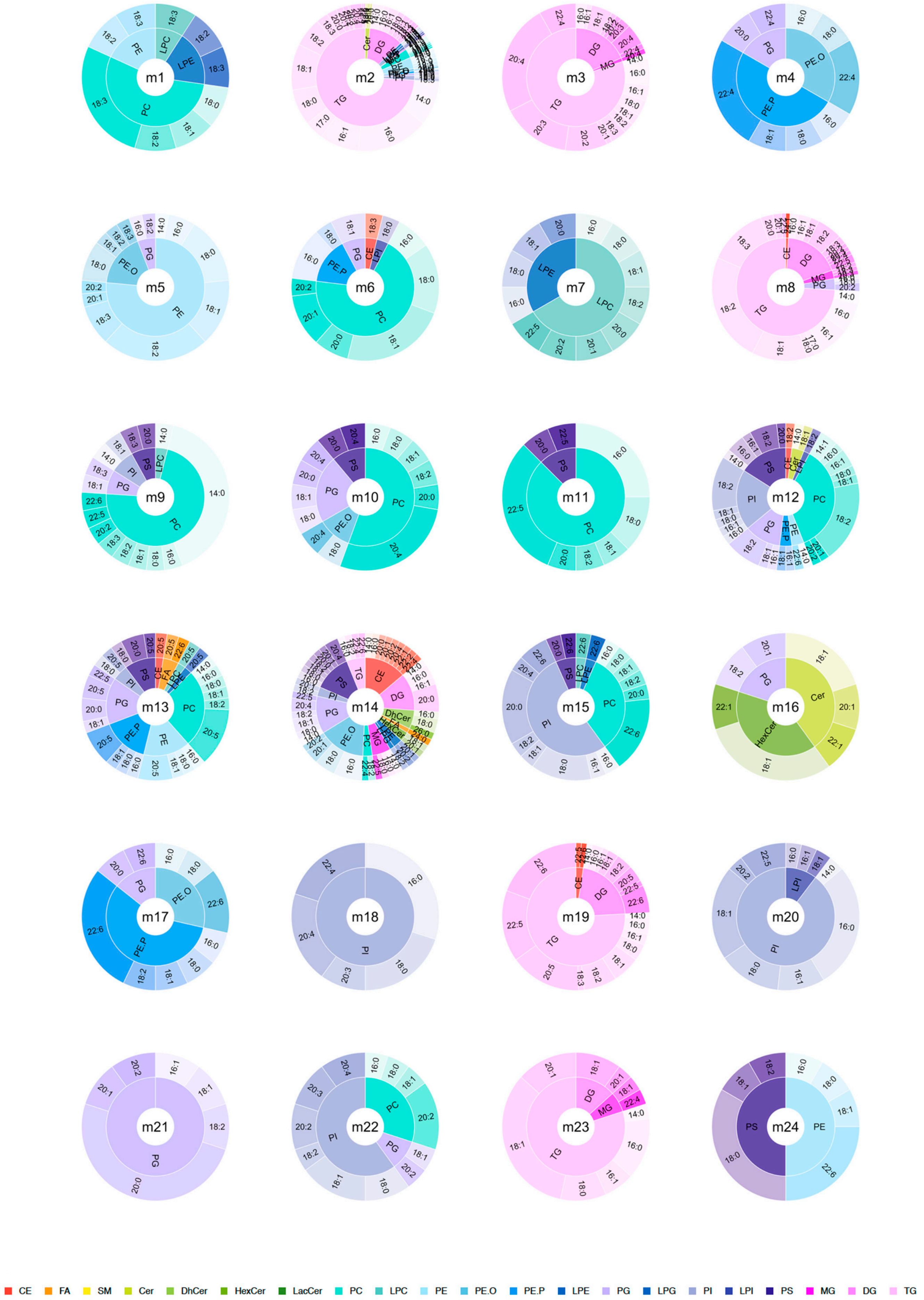
m1
m2
m3
m4
m5
m6
m7
m8
m9
m10
m11
m12
m13
m14
m15
m16
m17
m18
m19
m20
m21
m22
m23
m24
CE
FA
SM
Cer
DhCer
HexCer
LacCer
PC
LPC
PE
PE.O
PE.P
LPE
PG
LPG
PI
LPI
PS
MG
DG
TG

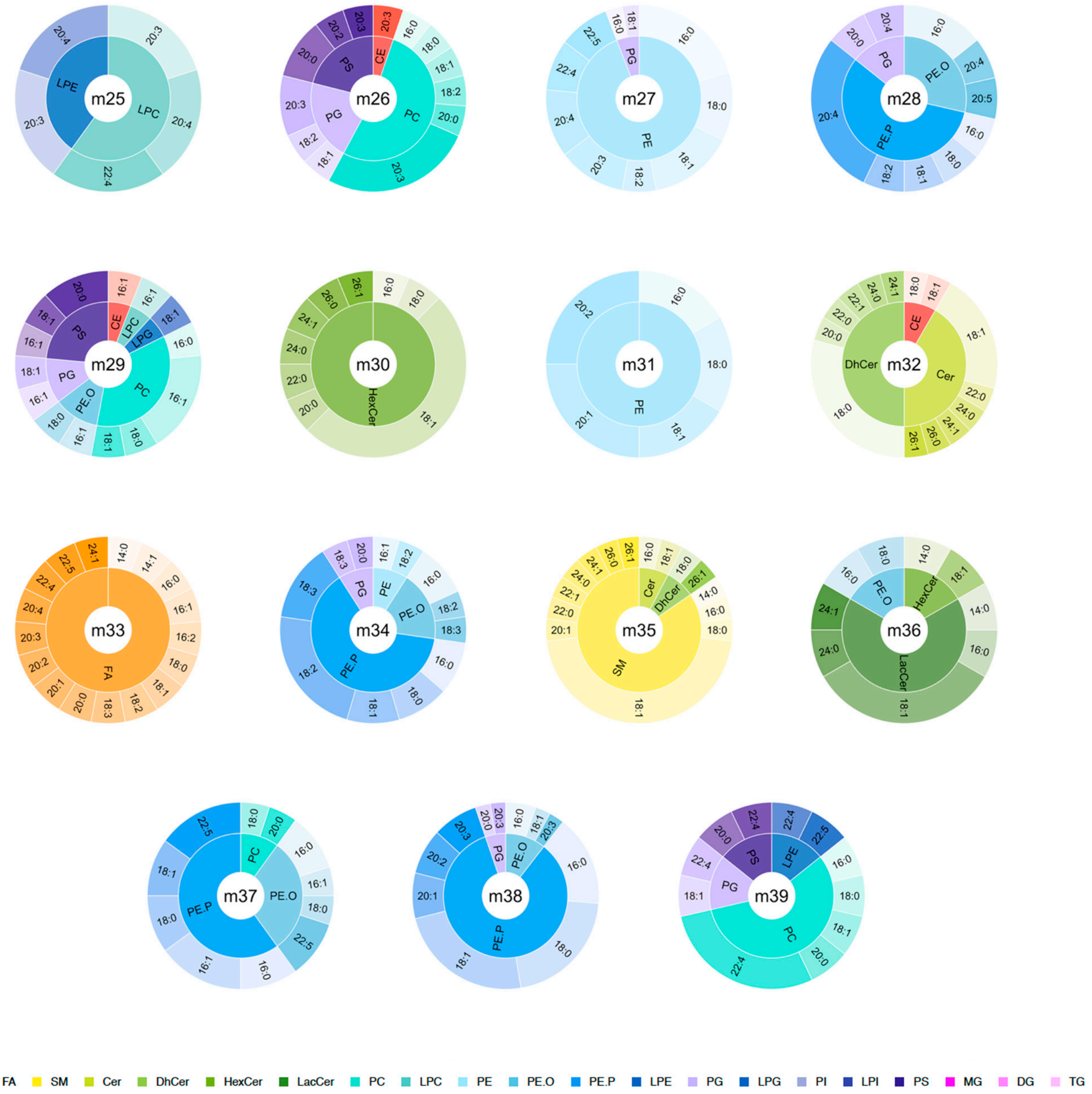

Figure S3 Pie-donut plots showing the proportional composition of lipid subclasses and fatty acyl chains within each WGCNA module. Each inner ring represents the relative abundance of lipid classes, while the outer ring shows the corresponding fatty acid chain distributions within that class. Colours correspond to lipid subclasses as indicated in the legend.
